# Pathology of healing: what else might we look at?

**DOI:** 10.1002/cam4.952

**Published:** 2016-10-26

**Authors:** Yoshiya Horimoto, Takuo Hayashi, Atsushi Arakawa

**Affiliations:** ^1^ Department of Breast OncologyJuntendo University School of MedicineTokyoJapan; ^2^ Department of Pathology and OncologyJuntendo University School of MedicineTokyoJapan; ^3^ Department of Human PathologyJuntendo University School of MedicineTokyoJapan

**Keywords:** Breast cancer, DCIS, healing, tumor infiltrating lymphocytes

## Abstract

Several aspects of the article by Morita et al. (Cancer Medicine 5:1607‐18, 2016), examining the spontaneous healing phenomenon with reference to tumor infiltrating lymphocytes (TILs), require clarification. The concept of “healing”, which can perhaps be more accurately termed “regressive change”, remains controversial due to a lack of concrete evidence. Since regressive change is characterized by fibrosis and lymphocytes, a cancer nest that appears to lack a distinct basement membrane, surrounded only by lymphocytes, as in Morita et al's Figure 2F, should be meticulously examined because the appearance may correspond to a tumor having just completed the process of invasion. In our experience, a layer of myoepithelial cells in such foci is often difficult to detect even with immunohistochemistry. Thus, we suggest evaluating the viability of cancer cells within the nest by employing several markers, such as Ki67 and apoptotic markers, to judge whether the tumor is intraductal. It might also be useful to compare cases with versus without regressive change to elucidate the biology of such tumors. For these reasons, a tumor, floating within a pool of TILs and lacking obvious fibrous bands, might be an interesting material to examine in future studies.

We read with interest the Morita et al. article 1, which described the spontaneous healing phenomenon of ductal carcinoma in situ (DCIS) with reference to tumor infiltrating lymphocytes (TILs), in an effort to elucidate the biology of DCIS and immune cells. While taking an interesting approach, several aspects of this study require clarification.

The “healing” concept has been recognized for more than 80 years 2. However, it has not yet been widely accepted by pathologists. “Healing”, which can perhaps be more accurately termed “regressive change” 3, 4, remains controversial due to lack of concrete evidence. The most uncomfortable aspect of this issue for many pathologists is a cancer nest, surrounded by lymphocytes with an apparent lack of basement membrane, being recognized as undergoing a process leading to cell death. Furthermore, this process is seemingly mediated by immune cells. Worryingly, however, this appearance of cancer cells may also correspond to a tumor having just completed the process of invasion. We regard this as a major concern, since the diagnosis and management would be completely different in these two circumstances.

As described by Hoda 5, we understand that the process of regressive change is characterized by fibrosis and infiltration of lymphocytes constituting an inflammatory reaction by the host. Thus, a cancer nest that appears to lack a distinct basement membrane, surrounded only by lymphocytes, as in figure 2F by Morita et al. 1, should be meticulously examined. Although Morita et al. stated that they confirmed the presence of myoepithelial cells by p63 immunostaining in foci of uncertain malignancy, in our experience, a layer of myoepithelial cells in such foci is often difficult to detect even when employing immunohistochemistry (IHC) for a panel of myoepithelial cell markers such as p63 and CD10. Thus, we suggest evaluating the viability of the cancer cells within the nest by employing several markers, such as Ki67 and apoptotic markers. If an intraductal lesion recedes during the process of regressive change, the cancer cells should show low Ki67 expression and a high number of apoptotic events, while the reverse would be true for invasive disease. IHC targeting cleaved‐Caspase 3 detects cancer cells undergoing apoptosis, even before the manifestation of morphological changes such as apoptotic body formation. Previous studies employed this method 6, 7, 8 and Pape‐Zambito et al. revealed a positive correlation between Ki67 and cleaved‐Caspase 3 in DCIS 6. We recently showed cleaved‐Caspase 3 to predict poor outcomes in patients who received neoadjuvant chemotherapy 9. Although surgical specimens after chemotherapy, quite different from DCIS, were used in that study, we believe that this protein might still be useful for evaluating DCIS. While establishing the viability of cancer cells as a method of judging whether a tumor remains intraductal requires well‐designed future studies, it might also be useful to compare cases with versus without regressive change to elucidate the biology of such tumors.

CD8‐positive T cells, which have a primary role in cellular immunity, might need to be in direct contact with cancer cells, rather than exerting their effects through thick fibrous bands. Humoral immunity, which involves mainly B cells, might become dominant once thick fibrous bands have formed. For these reasons, the TIL population surrounding a cancer nest may change in accordance with the observed stages of regressive change, as defined by the authors. Thus, TILs including CD8‐potisive T cells should be evaluated according to these stages. Focusing on TILs is very interesting as various studies have recently revealed numerous mechanisms of cancer immune‐editing. While strong TIL invasion has been reported to be related to better patient outcomes 10, 11, cancer cells themselves can reportedly induce an escape phase by means of gathering regulatory T cells 12.

A tumor, floating within a pool of TILs and lacking obvious fibrous bands, might well be the most interesting material to examine in future studies, for the reasons described above.

## Conflict of Interest

None declared.
